# Ceftazidime-assisted synthesis of ultrasmall chitosan nanoparticles for biofilm penetration and eradication of *Pseudomonas aeruginosa*

**DOI:** 10.1038/s41598-023-40653-0

**Published:** 2023-08-18

**Authors:** Xiaoran Zheng, Min Gao, Liangquan Wu, Xin Lu, Qiuqi Lin, Hai Zhong, Yingfei Lu, Yunlei Zhang, Xiuwei Zhang

**Affiliations:** 1 Department of Respiratory and Critical Care Medicine, The Affiliated Jiangning Hospital of Nanjing Medical University, Nanjing, 211100 China; 2 School of Biomedical Engineering and Informatics, Nanjing Medical University, Nanjing, 211100 China; 3 Central Laboratory, Translational Medicine Research Center, The Affiliated Jiangning Hospital of Nanjing Medical University, Nanjing, 211100 China

**Keywords:** Drug discovery, Diseases

## Abstract

*Pseudomonas aeruginosa* (*P. aeruginosa*) infections present a grave threat to immunocompromised individuals, particularly those with cystic fibrosis due to the development of bacterial biofilms. In this study, we engineered self-assembling chitosan-ceftazidime nanoparticles (CSCE) capable of effectively penetrating biofilms and eradicating *P. aeruginos*a. The CSCE nanoparticles were synthesized through ionic cross-linking, combining negatively charged ceftazidime with positively charged chitosan, resulting in uniform nanoparticles measuring approximately 40 nm in diameter, exhibiting high dispersity and excellent biocompatibility. Remarkably, these nanoparticles exhibited significant inhibition of *P. aeruginosa* growth, reduced pyocyanin production, and diminished biofilm formation, achieving a maximum inhibition rate of 22.44%. Furthermore, in vivo investigations demonstrated enhanced survival in mice with abdominal *P. aeruginosa* infection following treatment with CSCE nanoparticles, accompanied by reduced levels of inflammatory cytokines Interleukin-6 (125.79 ± 18.63 pg/mL), Interleukin-17 (125.67 ± 5.94 pg/mL), and Tumor Necrosis Factor-α (135.4 ± 11.77 pg/mL). Critically, mice treated with CSCE nanoparticles showed no presence of bacteria in the bloodstream following intraperitoneal *P. aeruginosa* infection. Collectively, our findings highlight the potential of these synthesized nanoparticles as effective agents against *P. aeruginosa* infections.

## Introduction

*Pseudomonas aeruginosa* (*P. aeruginosa*) infection poses a persistent and formidable challenge, particularly among patients with compromised immune systems or cystic fibrosis. In cystic fibrosis patients, the prevalence of *P. aeruginosa* can be as high as 47%^1^. Conventional treatment approaches involve the use of antibiotics, including third-generation cephalosporins (e.g., Ceftazidime), semi-synthetic penicillin (e.g., Piperacillin), and carbapenems (e.g., Meropenem)^2^. However, drug-resistant strains of *P. aeruginosa* have emerged, significantly compromising the therapeutic efficacy of these drugs. Mechanisms contributing to drug resistance include deficiencies in the OprD membrane protein, increased permeability of the outer membrane, active efflux systems, and overproduction of β-lactamase^3,4^. Moreover, the formation of *P. aeruginosa* biofilms, facilitated by the polysaccharide matrix, presents additional challenges by evading host immune responses and impeding drug penetration^5^. Various strategies targeting quorum sensing, iron metabolism, biofilm matrix formation, and bacterial colonization have been explored to combat *P. aeruginosa* biofilms, but satisfactory outcomes remain elusive^5^.

Recent studies have unveiled promising strategies to enhance the antibacterial efficacy against *P. aeruginosa*. Adjusting the ratio of hydrophilic primary ammonium salts to hydrophobic triphenylamine has demonstrated substantial antimicrobial effects by disrupting the bacterial membrane^6^. Introducing tryptophan and arginine into the antimicrobial peptide Jelleine-1 has proven effective against multi-drug-resistant *P. aeruginosa*^7^. Notable strategies to overcome drug resistance include photo-induced catalytic degradation activity of GO/Zn (Cu)O nanocomposites^8^, antimicrobial peptide TAT-RasGAP317-326^9^, and pH-sensitive surface charge-switchable azithromycin-encapsulated micelles^10^. However, their limitations in antimicrobial ability, production costs, hepatorenal toxicity, and stability hinder their progression to the preclinical stage^11^. Chitosan, a naturally occurring cationic glycan^12,13^ with large surface-to-volume ratio^14^, high functionalization possibilities and a greater capacity for drug loading^15,16^, exhibits high biocompatibility^17–19^ and biodegradability, making it an attractive candidate for various applications^20–22^. Chitosan-based materials, such as chitosan-agarose full polysaccharide silver nanocomposite films^23^ and chitosan-magnesium oxide nanoparticles^24^, have shown superior biofilm penetration, antibacterial activity, and antioxidant capabilities against *P. aeruginosa*. Chitosan-decorated graphene exhibits potent inhibitory effects on biofilm formation partially due to the chemical effect of graphene involving an oxidative stress and the physical effect related to the cell damage caused by the sharp edges of the graphene nanosheets^25^, opening avenues for potential use in implantable systems for urinary infection treatment^26^. Additionally, the inclusion of chitosan in nanocomposites of graphene and magnetite achieves highly efficient antibacterial activity against extended-spectrum β-lactamase-producing *P. aeruginosa*^27^. Chitosan hydrogel enhances the anti-inflammatory and antibacterial properties of chlorhexidine^28^. Furthermore, the development of ultra-high-sensitive V3.6Mo2.4O16-chitosan nanocomposites presents a promising avenue for electrochemical monitoring of hydroxychloroquine sulfate in environmental and clinical diagnostics^29^. Despite the advantages of chitosan-based materials, their production requires intricate processes and specific technologies. Moreover, efficient clinical drugs for immune deficiency or cystic fibrosis patients infected with *P. aeruginosa* remain elusive.

In this study, we aimed to develop a novel and straightforward method to prepare chitosan nanocomposites capable of penetrating biofilms and eradicating *P. aeruginosa*. Chitosan, known for its exceptional biocompatibility and biodegradability, has been extensively used in surgical applications and drug additives. Ceftazidime, a potent anti-*P. aeruginosa* drug, exhibits strong therapeutic effects against the bacterium. By dissolving chitosan in acetic acid, protonation of the –NH_2_ groups occurs through interaction with H^+^ ions, resulting in the formation of –NH_3_ moieties. The negatively charged ceftazidime then binds to the positively charged chitosan. Addition of TPP facilitates the binding of its P–O^−^ groups to the –NH_3_^+^ groups within the chitosan molecule, reducing chitosan hydration and promoting the formation of nanoparticles with a regular shape and suitable particle size. Importantly, the inclusion of ceftazidime reduces the binding of TPP to chitosan, resulting in smaller chitosan nanoparticles (approximately 40 nm) compared to those produced by ionotropic gelation (200–400 nm). Compatibility studies demonstrated that the nanocomposites exhibited high compatibility with red blood cells and mammalian cells (293 T cell line), with no observed toxicity upon intraperitoneal (i.p.) injection in healthy mice. Furthermore, the chitosan nanocomposites effectively reduced pyocyanin production, suppressed biofilm formation, and promoted biofilm degradation, ultimately eliminating *P. aeruginosa* from the abdominal cavity of infected mice. This was supported by decreased levels of interleukin-6 (IL-6), interleukin-17 (IL-17), and tumor Necrosis Factor-α (TNF-α). Leveraging the established clinical usefulness of chitosan and ceftazidime in treating bacterial infections, the nanocomposites developed in this study hold immense potential as highly effective treatment options for eradicating *P. aeruginosa* in external or systemic applications. This approach could be particularly beneficial in overcoming refractory *P. aeruginosa* infections in immunodeficient patients and expanding the clinical use of chitosan.

## Results

**Figure 1:**
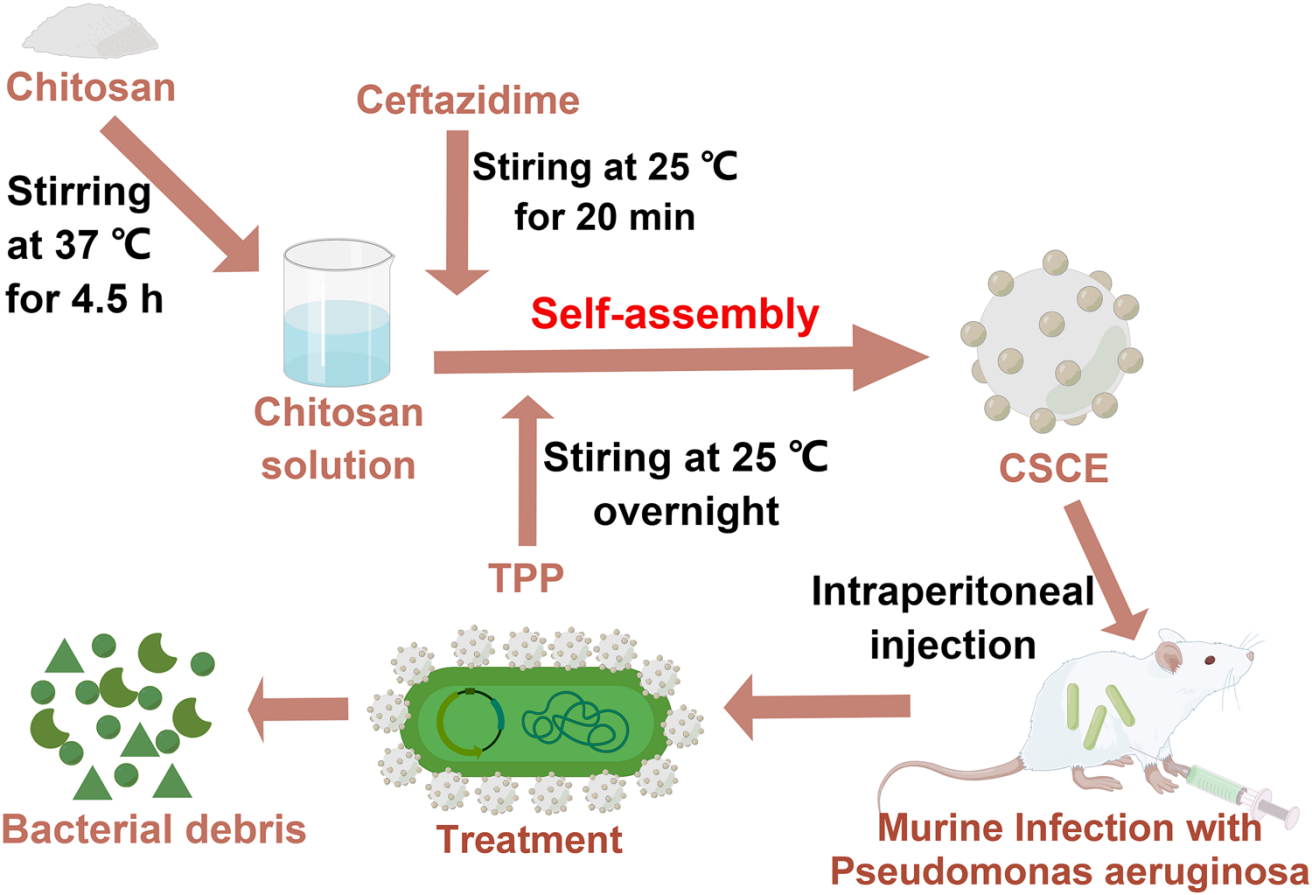
Figure legend of construction of CSCE nanoparticles for eradicating *P. aeruginosa* PAO1 infection from mouse abdominal cavity. To synthesize CSCE nanoparticles, ceftazidime (CE) was attached on chitosan nanoparticles under the catalysis of TPP through using the ionotropic gelation method. The resulted nanoparticles could exert the synergistic effects of chitosan and ceftazidime on the growth inhibition of *P. aeruginosa* PAO1. By Figdraw.

Figure 1 illustrates the fabrication procedure of chitosan-ceftazidime nanocomposites (CSCE) and their application in the eradication of *P. aeruginosa* from an intraperitoneally infected mouse. First, CSCE was prepared by adding ceftazidime during chitosan synthesis to exploit the electrostatic interaction between the two bioactive agents. The synthesis process took place in an aqueous solution by dissolving chitosan powder in 2% acetic acid liquid and adding TPP solution as a catalyst. This simple principle established the chitosan-derived nanoparticle as a versatile drug delivery system capable of transporting a range of negatively charged drugs for therapeutic purposes. The synergistic effects of chitosan and ceftazidime rendered the nanocomposites highly effective against *P. aeruginosa* infections. Furthermore, this delivery system could be used to deliver other drugs with negative charges.

**Figure 2:**
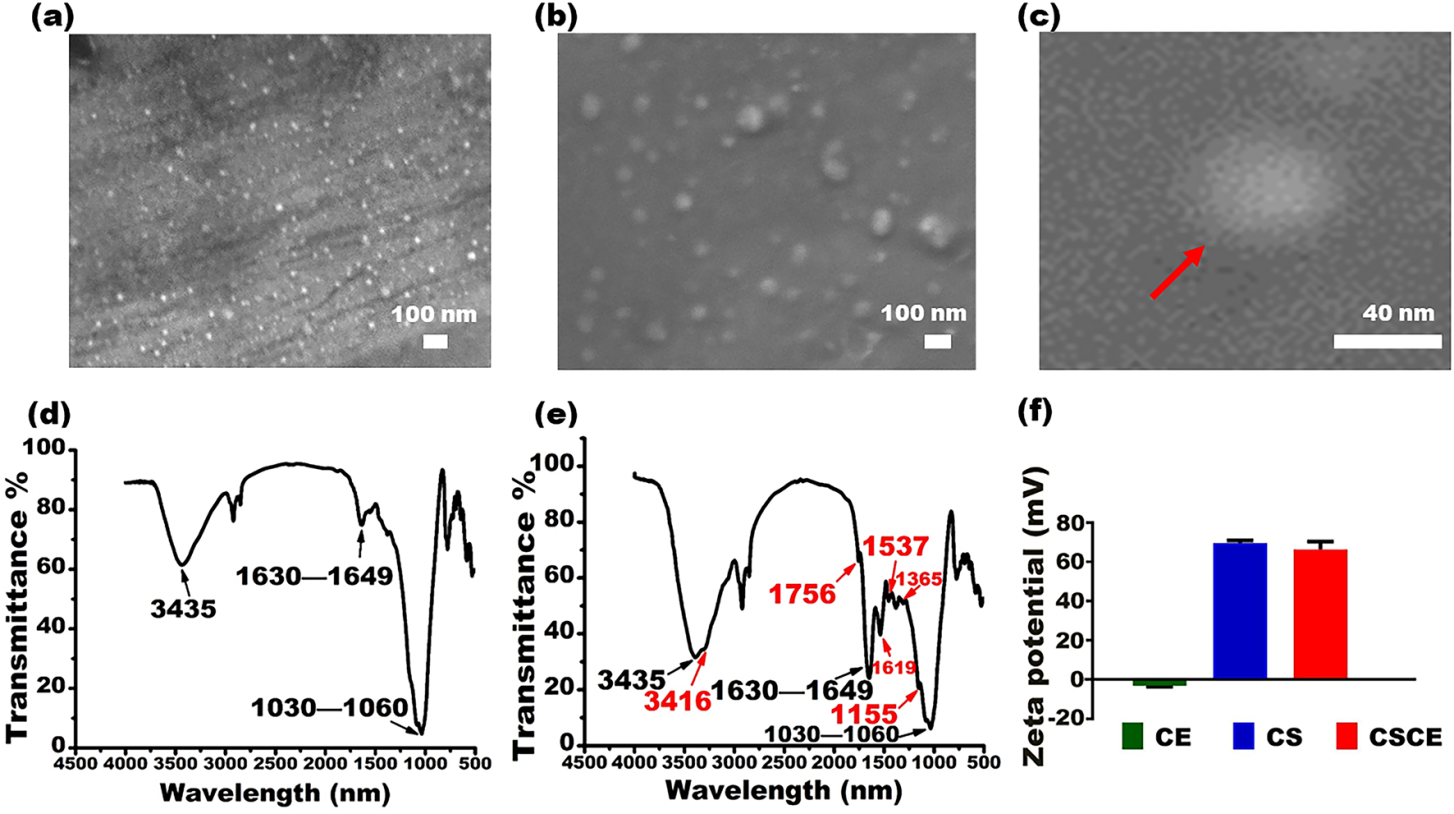
Characterization of the CSCE. (**a**) SEM image of CSCE. (**b**,**c**) Structural characteristics of CSCE at SEM magnification. (**d**) FT-IR spectrum of CS. (**e**) FT-IR spectrum of CSCE. (**f**) Zeta potentials of CE, CS, and CSCE.

Transmission Electron Microscope (TEM) and Scanning Electron Microscope (SEM) analysis revealed the morphology and size characteristics of chitosan-ceftazidime nanocomposites (CSCE), as depicted in Fig. 2a–c. The nanoparticles exhibited a uniform circular shape and displayed a narrow size distribution, with an average diameter of approximately 40 nm. Crucially, the CSCE nanocomposites demonstrated excellent dispersion, ensuring homogeneous distribution throughout the sample. The average diameter was determined to be 40.79 ± 6.83 nm, further confirming the consistent size profile of the nanocomposites. The Fourier Transform Infrared Spectroscopy (FT-IR) spectrum provided insights into the chemical composition of the CSCE nanocomposites. The characteristic absorption bands associated with C–O bonds were observed at 1030 cm^−1^ and 1060 cm^−1^. In addition, bending vibrations of the amide group were identified within the range of 1630–1649 cm^−1^, while stretching vibrations of NH_2_ and OH groups were detected around 3435 cm^−1^ (Fig. 2)^30^. These spectral features validate the successful synthesis of CSCE nanocomposites and the preservation of key functional groups. Significantly, the CSCE nanocomposites exhibited absorption bands corresponding to the characteristic vibrational modes of ceftazidime at 1756, 1619, 1537, 1365, 1155, and 3416 cm^−1^ (Fig. 2)^31^. These findings provide clear evidence of the successful loading of the drug onto the chitosan nanoparticles (CS), as illustrated in Fig. 2d,e. Furthermore, zeta potential measurements yielded values of − 3.18 mV, 69.66 mV, and 66.50 mV for CE (ceftazidime), CS, and CSCE nanoparticles, respectively (Fig. 2e). These results confirm the successful loading of ceftazidime onto the positively charged surface of the CS nanoparticles. Remarkably, the CS nanoparticles exhibited a loading capacity of up to 40 mg of ceftazidime per gram. This finding underscores the impressive drug loading capacity of the CSCE nanocomposites. SEM images of CSCE nanoparticles revealed that the inclusion of ceftazidime did not adversely affect the structure or morphology of the nanoparticles (Fig. 2a–c). These observations confirm the preservation of the nanoparticles' integrity following drug decoration. The comprehensive results obtained demonstrate the potential of well-dispersed and appropriately sized CSCE nanocomposites as effective drug candidates for combating *P. aeruginosa* infections. The uniform circular shape, consistent size distribution, successful drug loading, and structural stability observed collectively underscore the promising antimicrobial properties of these nanocomposites.

**Figure 3:**
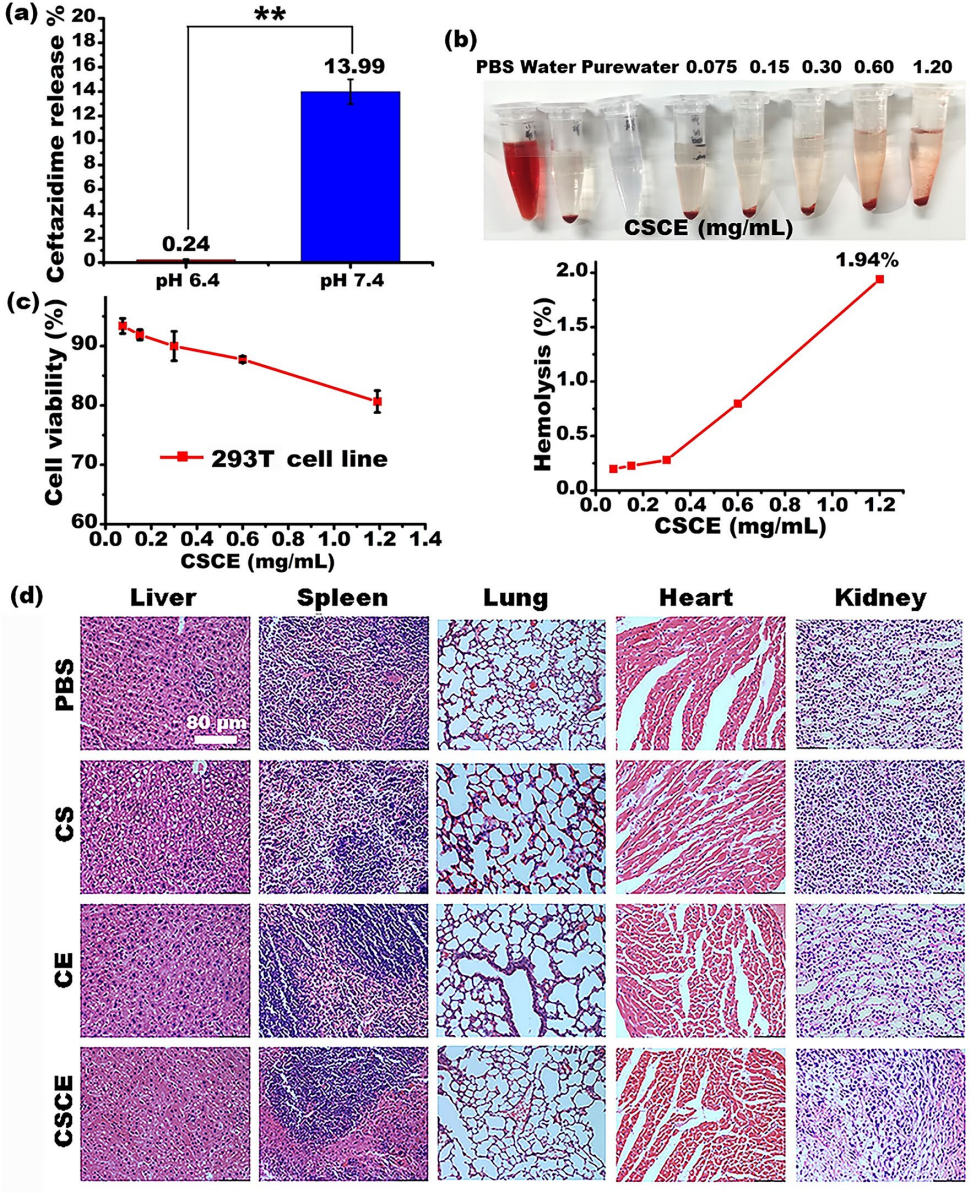
(**a**) Ceftazidime-releasing profiles of CSCE in the pH 6.4 and pH 7.4 solutions. (**b**) Hemolysis assay. The CSCE nanoparticles at different concentrations of 0.075, 0.15, 0.30, 0.60, and 1.19 mg/mL were incubated with RBCs at 37 °C for 2 h. The amount of Hemoglobin released from the RBCs was calculated through measuring the absorbance of the supernatants in a microplate reader at 490 nm. (**c**) Cytotoxicity of CSCE against 293 T cells. (**d**) H&E staining of the key organs from the healthy ICR mice (n = 3). *P < 0.05; **P < 0.01; The data represent the mean ± SD.

The effective release of ceftazidime within the targeted microenvironment plays a crucial role in the antibacterial activity of CSCE nanocomposites, while also minimizing potential side effects. *P. aeruginosa* biofilms are known to exhibit an acidic environment (pH 6.4 ± 0.2) that gradually decreases towards the biofilm core (pH 6.1 ± 0.3)^32,33^. Accordingly, the release profiles of ceftazidime from CSCE nanoparticles were investigated under acidic and neutral pH conditions. The results demonstrated notable differences in the release rates of ceftazidime from CSCE nanoparticles between acidic and neutral pH environments. Figure 3a presents the release profiles of ceftazidime after 24 h of incubation at 37 °C in pH 6.4 phosphate buffered saline (PBS) solution and pH 7.4 solution. Remarkably, the release rate of ceftazidime from CSCE nanoparticles reached as high as 13.99% in pH 6.4 PBS solution, while remaining significantly lower at 0.24% in pH 7.4 solution. This disparity can be attributed to the proximity of pH 6.4 to the optimal dissolution pH of ceftazidime (pH 6.0), facilitating the rapid detachment of the drug from the CSCE nanoparticles. The acid-dependent release properties of ceftazidime from CSCE nanocomposites offer several benefits. Firstly, it prevents undesired drug leakage in neutral body fluids, ensuring that the therapeutic agent remains intact until reaching the targeted site. Additionally, the pH-responsive release characteristics enhance drug release specifically when the nanoparticles are in contact with *P. aeruginosa* biofilms, enhancing their antibacterial efficacy. These findings highlight the ability of CSCE nanocomposites to selectively release ceftazidime in the acidic microenvironment of biofilms, maximizing the therapeutic impact while minimizing potential adverse effects.

To assess the suitability of CSCE for potential applications, their toxicity was preliminarily evaluated using in vitro methods. A Hemolysis assay, a standard approach for assessing the biocompatibility of drugs with red blood cell (RBC) membranes, was conducted. Figure 3b depicts the results, showing that hemoglobin levels slightly increased with increasing CSCE nanoparticle concentration. However, even at the maximum concentration of 1.2 mg/mL, the increase did not exceed 1.94% after 2 h of incubation, indicating the excellent biocompatibility of CSCE nanoparticles with RBCs. Furthermore, a cell cytotoxicity assay was performed to evaluate the impact of CSCE nanoparticles on 293 T cell growth. Figure 3c demonstrates that cell viability remained above 80% at all tested concentrations, except when the concentration exceeded 1.2 mg/mL. This finding highlights the high biocompatibility of the nanoparticles. In contrast, some studies reported lower cell viability (below 80%) when treating cells with their biomimetic amphiphilic chitosan nanoparticles at a concentration of 0.5 mg/mL^34^, which is lower than the concentration used in our study (1.2 mg/mL). It is important to note that the cytotoxicity observed at high CSCE nanoparticle concentrations may be attributed to physical obstruction of cell metabolism and biological processes due to a large quantity of nanoparticles attached to the cell membrane surface. To further evaluate the potential toxicity of CS nanoparticles and CSCE nanoparticles in vivo, assays were conducted using ICR mice. The mice (n = 8) were divided into groups and received i.p. administration of PBS, ceftazidime (CE), CS, or CSCE once weekly for one month. Throughout the experiment, no visible anaphylactic reactions or irregular behavior were observed in the mice. Additionally, key organs were harvested and subjected to H&E staining after one month (n = 3) to assess potential nanoparticle toxicity. As shown in Fig. 3d, i.p. administration of CS and CSCE did not induce any noticeable pathological effects on the organs of the ICR mice at the tested concentrations, affirming the feasibility of utilizing these nanoparticles for *P. aeruginosa* treatment.

**Figure 4:**
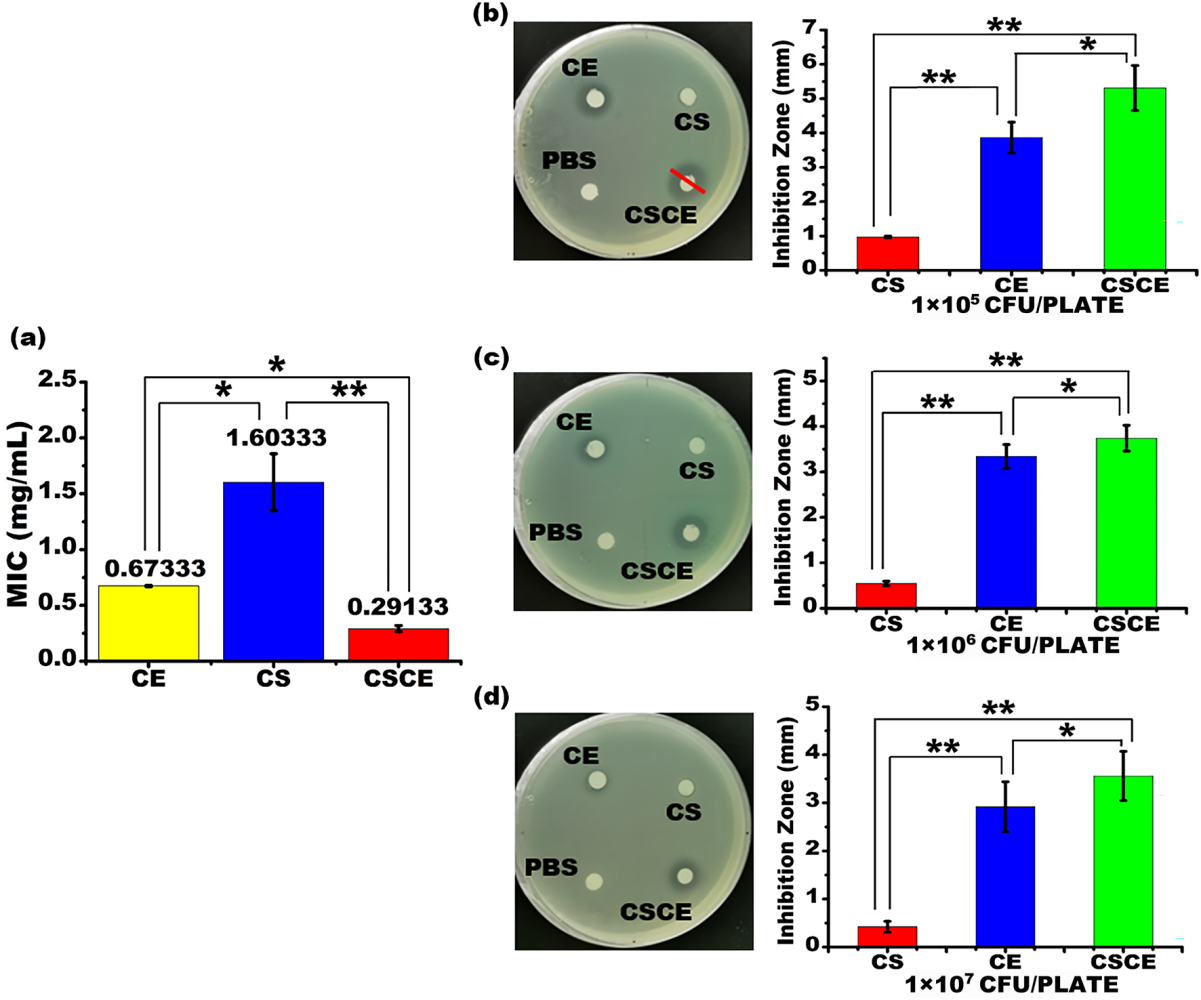
(**a**) Minimal Inhibitory Concentration Test of CS, CE, and CSCE against *P. aeruginosa* PAO1. (**b**–**d**) Antibacterial activity of CSCE nanoparticles against *P. aeruginosa* PAO1. Disk diffusion test of CE, CS, and CSCE using LB plates containing *P. aeruginosa* PAO1 at the concentrations of 1 × 10^5^, 1 × 10^6^, 1 × 10^7^ CFU/plate. *P < 0.05; **P < 0.01. The data indicate the mean ± SD.

Furthermore, we assessed the antibacterial properties of CE, CS, and CSCE nanoparticles using dilution susceptibility tests and Kirby–Bauer tests, following the established protocols. The minimal inhibitory concentration (MIC) values against *P. aeruginosa* PAO1 were determined for each nanoparticle: CE (0.673 mg/mL), CS (1.603 mg/mL), and CSCE (0.291 mg/mL), as illustrated in Fig. 4a. Notably, CSCE nanoparticles exhibited the lowest MIC value, indicating their enhanced efficacy likely resulting from synergistic effects between CS and CE. Our findings reveal that CSCE nanoparticles possess a relatively lower MIC value compared to previous investigations involving chitosan nanocomposites^35^. Moreover, the Kirby–Bauer test, conducted after an 18-h incubation at 37 °C, further substantiated the inhibitory effects of CSCE nanoparticles on *P. aeruginosa* PAO1 growth. As depicted in Fig. 4b–d, CSCE nanoparticles displayed the most pronounced inhibitory effects among the tested drugs. This superior performance can be attributed to the synergistic actions of CS and CE against *P. aeruginosa* PAO1. Additionally, it is worth noting that the inhibitory zone produced by the CS group was significantly smaller than that of the CE-treated group. This observation suggests that chitosan's antibacterial effect primarily stems from its ability to enhance the permeability of the negatively charged cell membrane, facilitated by the positive charge of chitosan nanoparticles. Conversely, CE directly inhibits cell wall synthesis. Taken together, our results demonstrate that CSCE nanoparticles successfully harness the antibacterial properties of chitosan and ceftazidime, effectively impeding the proliferation of *P. aeruginosa* PAO1. Furthermore, the combination of ceftazidime with CS nanoparticles manifests a more potent antimicrobial effect than either CS nanoparticles or ceftazidime alone. Therefore, our study underscores the promising potential of CSCE nanoparticles in augmenting the therapeutic efficacy of antimicrobial treatments, highlighting the significance of combination therapy in combating bacterial infections.

**Figure 5:**
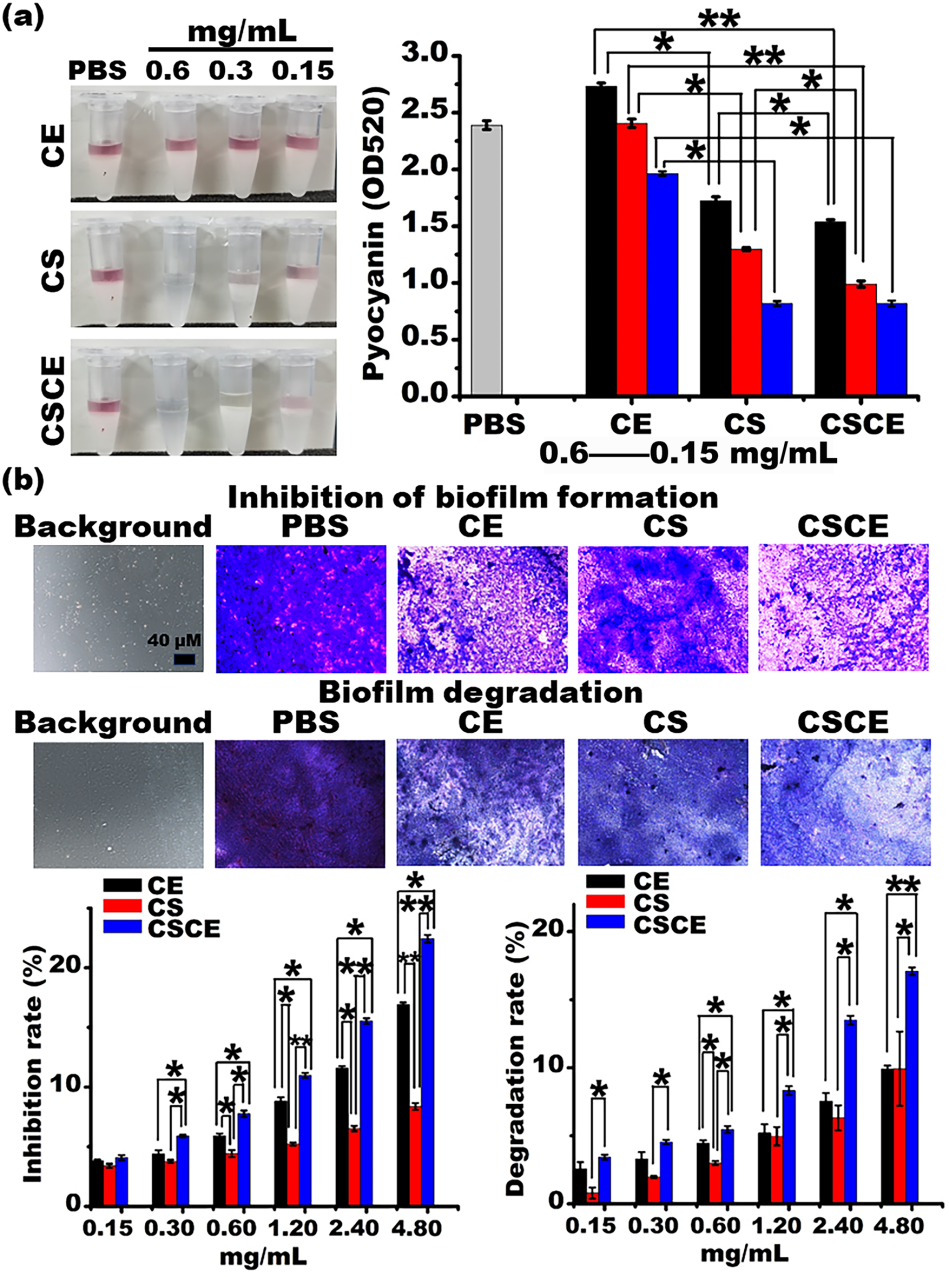
(**a**) Pyocyanin assay. (**b**) Restraining and degrading the biofilm formed by *P. aeruginosa* PAO1. PAO1 was incubated with different concentrations of PBS, CS, CE, and CSCE for 48 h at 37 °C in a 96-well plate. The biofilm formed by the bacteria was determined. *P < 0.05; **P < 0.01; the data represent the mean ± SD.

*Pseudomonas aeruginosa*, a known producer of pyocyanin, a redox-active secondary metabolite with zwitterionic properties, possesses the ability to easily permeate biological membranes^36^. Previous studies have demonstrated that pyocyanin can inactivate catalase and modulate Glutathione redox cycling in alveolar and bronchial epithelial cells^37^. In our investigation, we observed a significant concentration-dependent inhibition of pyocyanin production in *P. aeruginosa* upon treatment with CS and CSCE nanoparticles, as evidenced by the results of the pyocyanin quantification assay depicted in Fig. 5a. It is important to note that pyocyanin-deficient strains of *P. aeruginosa* have been shown to exhibit reduced cytotoxicity towards neutrophils^38^. This observation provides a possible explanation for the effective antibacterial effects of CSCE nanoparticles against *P. aeruginosa*. Inhibition of pyocyanin production represents an alternative therapeutic strategy for treating *P. aeruginosa* infections^39^, which may account for the superior antibacterial efficacy displayed by CSCE nanoparticles against *P. aeruginosa* PAO1. Furthermore, the production of pyocyanin was substantially lower in the CSCE group compared to the CS and CE-treated bacterial groups.

**Figure 6:**
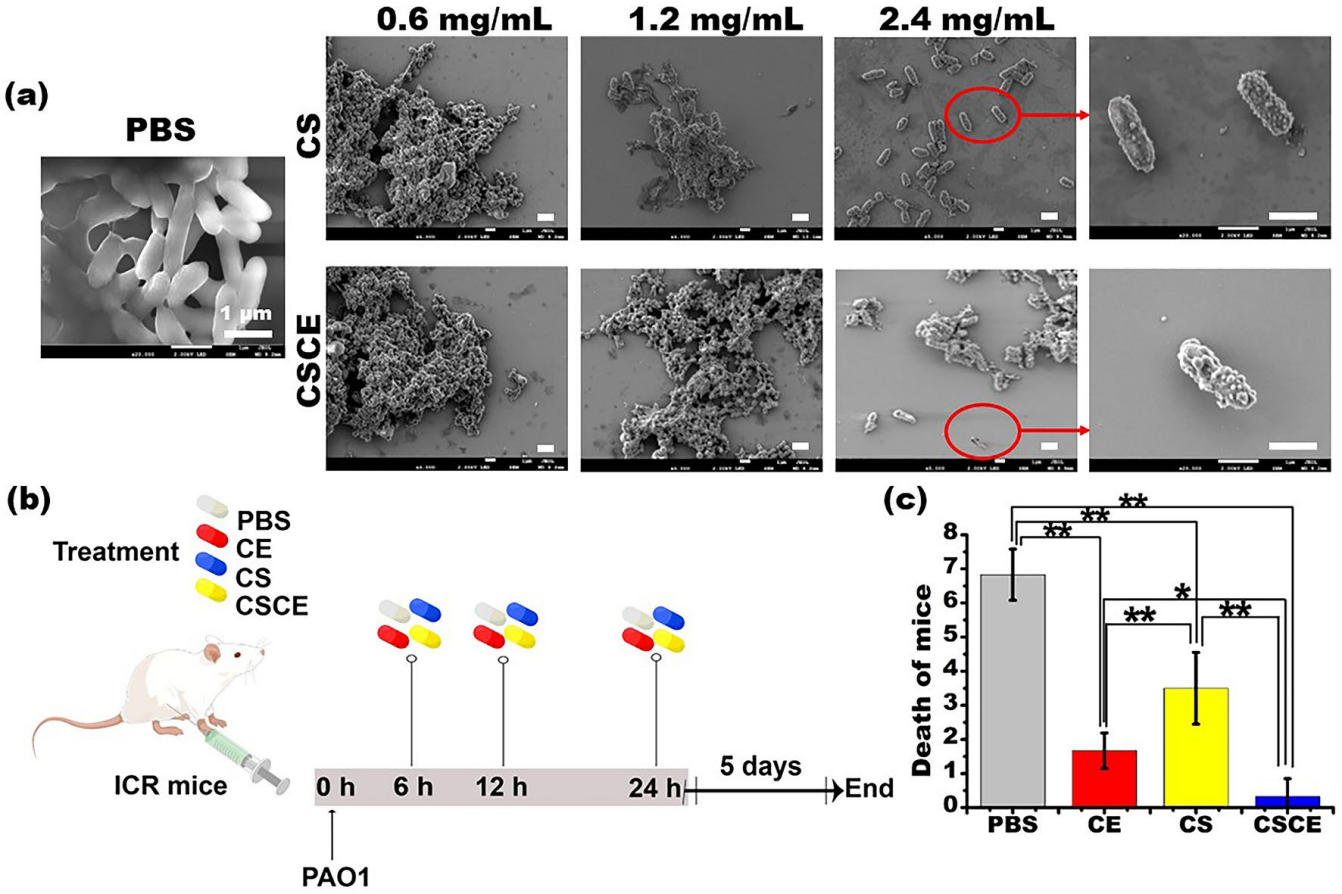
(**a**) SEM images of biofilms formed by *P. aeruginosa* PAO1 after incubation with different concentrations of CS and CSCE for 24 h at 37 °C in a shaking incubator. (**b**) Plot of the in vivo evaluation of the antibacterial effects of the CSCE nanocomposites. The abdomen-infected mice were i.p. administrated with PBS, CE, CS, CSCE at 6, 12, and 24 h after the establishment of *P. aeruginosa* PAO1 in the mouse abdomen. (**c**) The number of dead mice in these groups.

Biofilm formation plays a pivotal role in the development of drug resistance in *P. aeruginosa* infections^40^. Our study investigated the impact of CE, CS, and CSCE nanoparticles on biofilm formation of *P. aeruginosa* PAO1 in a concentration-dependent manner, comparing the results to the PBS control group (Fig. 5b). Remarkably, all of them demonstrated significant inhibition of biofilm formation. CSCE nanoparticles exhibited the strongest inhibitory activity, with an inhibition rate of 22.44% at a concentration of 4.8 mg/mL. CE and CS nanoparticles also displayed reductions in biofilm formation, with inhibition rates of 16.92% and 8.38%, respectively, at the maximum concentration tested. Biofilm is primarily composed of bacteria and an extracellular matrix^41^, and the decrease in bacterial load likely contributed to the reduction in biofilm formation. Additionally, we investigated whether CSCE, CS, or CE possessed the ability to degrade established biofilms formed by *P. aeruginosa* PAO1. Our findings demonstrated that CSCE nanoparticles exhibited the highest degree of biofilm degradation, reaching up to 17.09% at the maximum concentration (Fig. 5b). In contrast, the biofilm degradation rates in the CS and CE groups were comparatively lower, at 9.6% and 10.1%, respectively. Notably, there was no significant difference in biofilm degradation between the CE and CS groups at the maximum concentration (*P* > 0.05) (Fig. 5b). The antibacterial activity of CE likely contributed to its ability to degrade biofilms, while the positive charge of CS molecules facilitated binding to alginate, a major component of biofilms^42^. To gain further insight into the interaction between CSCE nanoparticles and bacteria, we employed SEM. As depicted in Fig. 6a, bacteria treated with PBS appeared to be enveloped by a "cell membrane." In contrast, *P. aeruginosa* PAO1 treated with CSCE nanoparticles exhibited an abundance of microparticles coating the bacteria. SEM images revealed that the *P. aeruginosa* PAO1 bacteria treated with CSCE nanoparticles exhibited a loose bacterial cluster, which became more pronounced with increasing nanoparticle concentration, indicating both antibacterial activity and biofilm degradation. Taken together, these findings suggest that CSCE nanoparticles possess the potential to inhibit biofilm formation, dismantle established biofilms, and eradicate bacteria. These results provide a foundation for future in vivo assays, highlighting the promise of CSCE nanoparticles as a therapeutic strategy against biofilm-associated *P. aeruginosa* infections.

**Figure 7:**
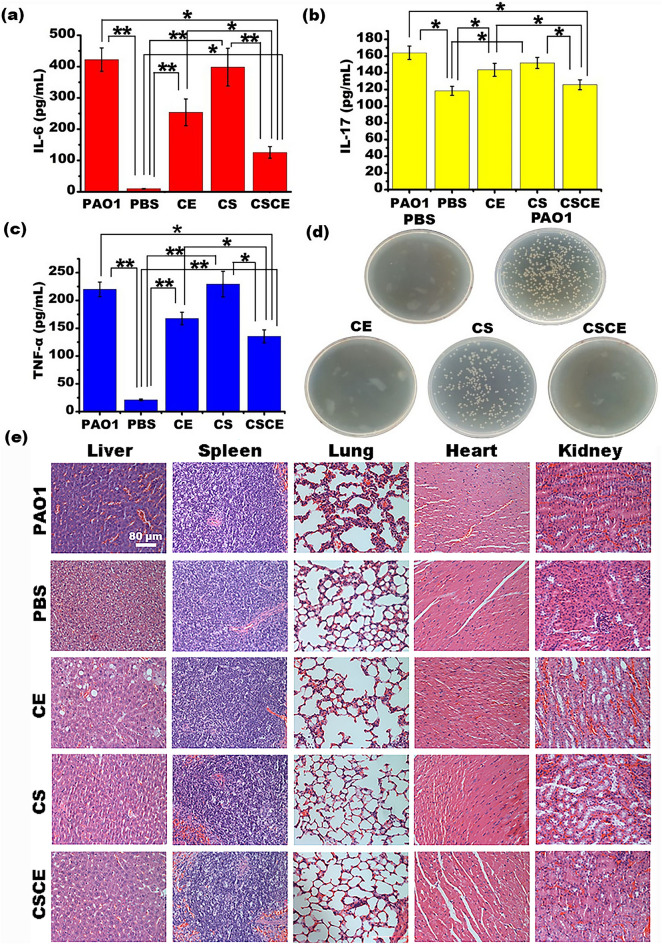
IL-6 (**a**), IL-17 (**b**), TNF-α (**c**) in the mice lungs through ELISA. (**d**) The colony numbers of *P. aeruginosa* PAO1 from lung homogenate on the Luria–Bertani (LB) plates. (**e**) The pathological characteristics of the mice were analyzed via the H&E staining (n = 5). The control (PBS) group indicates that the healthy mice in the group did not receive any bacterial infection or treatment.

Furthermore, our study aimed to assess the effectiveness of CSCE nanoparticles in eradicating *P. aeruginosa* PAO1 in an abdominal infection model using ICR mice. The mice were randomly divided into five groups: PBS, PAO1, CE, CS, and CSCE. Intraperitoneal administration of PBS, CE, CS, and CSCE nanoparticles was initiated at 6, 12, and 24 h after *P. aeruginosa* PAO1 infection, as illustrated in Fig. 6b. On the fifth day following bacterial infection, a substantial number of mice in the PBS and CS groups succumbed to the infection, as demonstrated in Fig. 6c. The remaining mice were sacrificed, and their bronchoalveolar lavage fluids (BALF) were collected to quantify the cytokines interleukin-6 (IL-6), interleukin-17 (IL-17), and tumor necrosis factor-α (TNF-α). Figure 7a presents the concentration of IL-6 in the *P. aeruginosa* PAO1 group, which reached 422.34 ± 37.41 pg/mL after five days. In contrast, the concentrations in the PBS, CE, CS, and CSCE groups were measured to be 9.8 ± 0.92, 253.65 ± 42.67, 398.21 ± 60.23, and 125.79 ± 18.63 pg/mL, respectively. Additionally, IL-17 concentrations were notably lower in the CE, CS, and CSCE groups, ranging from 143.52 ± 7.75 to 125.67 ± 5.94 pg/mL, compared to the mice infected with PAO1 (163.93 ± 8.05 pg/mL) (Fig. 7b). Similarly, the TNF-α test yielded consistent results, with concentrations in the PBS and CSCE groups measuring 21.27 ± 1.62 and 135.4 ± 11.77 pg/mL (n = 3), respectively. These values were significantly lower than those in the PAO1 group (219.97 ± 13.19 pg/mL) (n = 3). The CE and CS groups exhibited concentrations of 167.57 ± 11.28 and 229.43 ± 23.12 pg/mL, respectively (n = 3) (Fig. 7c). In summary, our findings conclusively demonstrate that CSCE nanoparticles possess therapeutic potential in combating *P. aeruginosa* PAO1 infections through their regulation of cytokine responses.

Our findings present compelling evidence for the protective efficacy of CSCE nanoparticles against *P. aeruginosa* PAO1 infection in mice. Specifically, the bacterial culture of lung homogenates from the CE and CSCE treatment groups exhibited a minimal presence of bacteria, resembling the levels observed in the blank control groups (Fig. 7d). Furthermore, histological examination through H&E staining revealed that the alveolar septum in the PAO1 group appeared congestive and thickened, while the organs of mice in the CE, CS, and CSCE groups remained largely unaffected, akin to those in the control group (Fig. 7e). Importantly, i.p. administration of CE, CS, and CSCE nanoparticles did not induce any discernible pathological effects on the isolated tissues of ICR mice at the tested concentrations. The observed pathological changes in the lung tissues of the PAO1 group were indicative of bacteremic sepsis resulting from the i.p. injection of *P. aeruginosa* PAO1. Upon injection into the left abdomen, *P. aeruginosa* PAO1 dissemination impacted alveolar perfusion, a preliminary injury manifestation of sepsis^43^. Over subsequent days following PAO1 injection, lung injury, such as congestion, became evident. However, the administration of CSCE nanoparticles mitigated this damage and attenuated infection among the mice. Hence, our results highlight the potential utilization of CSCE nanoparticles for in vivo eradication of *P. aeruginosa* PAO1.

## Discussion

Here, we present a novel approach for the fabrication of ultrasmall chitosan nanoparticles containing the potent antimicrobial drug, ceftazidime, with the aim of effectively treating *P. aeruginosa* PAO1 biofilm infections. The engineered CSCE nanoparticles with an average diameter of 40 nm demonstrated remarkable hemocompatibility, as evidenced by a maximum hemolysis rate of merely 1.94%. Furthermore, these nanoparticles exhibited low toxicity towards normal cells, with a minimum viability of 80%. The efficacy of the CSCE nanoparticles was demonstrated through their ability to inhibit the growth of *P. aeruginosa* PAO1, suppress pyocyanin production, impede biofilm formation, and degrade pre-existing biofilms. Notably, upon i.p. injection, the CSCE nanoparticles significantly reduced the mortality of the ICR mice suffering from *P. aeruginosa* infection. Given the broad-spectrum antibacterial properties of chitosan and the established pharmaceutical utility of ceftazidime, the CSCE nanoparticles hold immense potential as a delivery system for various functional molecules, enabling the effective combat of other pathogenic bacteria.

In this study, we have successfully fabricated a novel chitosan-based nanocomposite using a streamlined one-step method. This significant accomplishment brings forth several key advantages to the system, including a remarkable reduction in the particle size of chitosan nanoparticles (down to an impressive 40 nm), enhanced physicochemical stability, controlled biotoxicity, and improved drug efficacy. Chitosan, being a natural polymer due to high number of active sorptive sites and high content of –NH_2_ and –OH groups, which can reduce the leakage of metallic and organic compounds into aqueous solution, exhibits the remarkable ability to delay cellular uptake of ceftazidime when tethered to its surface^44^, thereby extending the drug's action time, avoiding the aggregation of nanoparticles and bolstering its antimicrobial potency due to their rational design^45^. Moreover, the integration of chitosan with ceftazidime in a single step underscores the efficiency and economy of this approach. Furthermore, our investigation delves into the role of pyocyanin, a well-known extracellular electron carrier that employs nicotinamide adenine dinucleotide (NADH) as its initial endogenous reductant, with molecular oxygen serving as the terminal electron acceptor^46^. Pyocyanin is also recognized for its ability to reduce ferric ions to the soluble ferrous state, which can be readily utilized by *P. aeruginosa* to meet its nutritional requirements^47^. Despite the significance of pyocyanin as one of the main virulence factors of* P. aeruginosa*, its relationship with the antimicrobial properties of chitosan nanoparticles has been relatively understudied. Here, we have taken an innovative approach by investigating the impact of CSCE nanoparticles on pyocyanin production. Intriguingly, our findings reveal a significant reduction in pyocyanin production within the CSCE group, thereby highlighting the potent anti- *P. aeruginosa* capabilities of CSCE nanoparticles. This novel discovery contributes to expanding our understanding of the antimicrobial properties of chitosan nanoparticles and underscores their potential in combating *P. aeruginosa* infections.

Chitosan nanoparticles can be produced using various techniques, such as polymerization of chitosan with methacrylic acid, reverse micelle medium, or microemulsion methods^48^. For instance, Rasoulzadehzali et al. prepared a chitosan-based bio-nanocomposite for antibiotic delivery, but the reaction temperature required was as high as 80 °C^49^. Wilson et al. developed chitosan-functionalized nanoparticles through polyelectrolyte adsorption, which involved complex steps^50^. In contrast, our method for fabricating the chitosan composite utilizes commonly available experimental apparatus, simple formulation, and mild experimental conditions. Notably, the size of our chitosan nanoparticles is a mere 40 nm, significantly smaller than similar nanoparticles prepared using ionotropic gelation^51^, as well as other chitosan-based nanoparticles such as carvacrol-chitosan nanoparticles^52^ and chitosan-poly-ε-caprolactone (CS_PCL) nanoparticles^53^. Nanoparticles smaller than 100 nm offer several advantages, including improved patient compliance, enhanced biodistribution, and site-specific drug delivery^54^, especially for internal administration. Furthermore, smaller nanoparticles have demonstrated advantages in reducing cellular uptake by the mononuclear phagocytic system, prolonging their circulation time in the bloodstream, and facilitating cellular penetration, ultimately leading to improved antibacterial efficacy. There have also been reports highlighting the superior capillary penetration and tissues permeability of smaller nanoparticles^55^.

Despite the remarkable properties of our nanocomposite, including its small diameter, excellent anti-*P. aeruginosa* efficacy, and biocompatibility, further work is required to enhance its potential and facilitate its translation into clinical applications. There are several important aspects that our team intends to address. Firstly, it is crucial to evaluate the therapeutic effectiveness of CSCE nanoparticles in a human infection model. This would provide valuable insights into their performance and efficacy in a relevant clinical setting, allowing us to assess their suitability for further development. Secondly, to expand the versatility of our method for fabricating antibiotic-chitosan nanocomposites, it is necessary to explore the combination of additional antimicrobial molecules. This will enable us to investigate the efficacy and synergistic effects of the nanocomposite against a broader range of pathogens, thus enhancing its potential as a multi-functional therapeutic platform. Furthermore, considering the potential clinical usage, it is essential to conduct further assays to assess the safety and effectiveness of CSCE nanoparticles over an extended treatment course. Additionally, it is important to investigate the potential interactions between CSCE nanoparticles and the immune system to better understand their immunological impact and ensure their overall compatibility.

In conclusion, our study presents a novel strategy for effectively eliminating *P. aeruginosa* biofilms. This drug carrier significantly enhances the therapeutic effects of antibiotic molecules, offering promising prospects in the treatment of challenging bacterial infections. This significant achievement not only holds implications for the clinical use of chitosan-based nanocomposites in combating other diseases such as cancer but also highlights the broader applicability of natural materials like chitosan. Furthermore, the successful integration of chitosan, a natural material, emphasizes the broader use of such environmentally friendly substances in diverse biomedical applications. Overall, this study contributes to advancing our understanding of biofilm eradication and drug delivery strategies. It signifies the potential of chitosan-based nanocomposites as transformative therapeutic agents, paving the way for future research and clinical exploration in a range of diseases and therapeutic approaches.

## Materials and methods

### Materials and chemicals

Chitosan (deacetylation degree is higher than 75%) and 0.1% (w/w) TPP were purchased from Shanghai Aladdin Biochemical Technology Co., Ltd. 2% (w/w) Acetic Acid, and Crystal violet staining solution were obtained from Sangon Biotech (Shanghai) Co., Ltd. Ceftazidime was acquired from Sinopharm Zhijun (Shenzhen) pharmaceutical Co., Ltd (Shenzhen, China). *P. aeruginosa* was bought from China General Microbiological Culture Collection Centre. Glutaraldehyde and paraformaldehyde were provided by SenBeiJia Biological Technology Co., Ltd.), Anhydrous ethanol, Chloroform and 0.2 H Hydrochloric acid (HCl) were bought from Nanjing Chemical Reagent Co., Ltd. Dulbecco’s modified Eagle’s medium (DMEM) and 0.05% trypsin-ethylenediaminetetraacetic acid (EDTA) were purchased from Nanjing Keygen Biotech Co., Ltd. (Nanjing, China), Cell Counting Kit-8 (CCK-8) was from Dojindo Chemical Technology (Shanghai) Co., Ltd. Pentobarbital Sodium was purchased from Merck Pharmaceutical Manufacturing (Jiangsu) Co. Ltd. ICR mice were purchased from Jiangsu Laboratory Animal Centre of Nanjing Medical University. The study was conducted in accordance with the Declaration of Helsinki, and approved by the Animal Ethics Committee of Nanjing Medical University (IACUC 2303018). We confirm that all experiments were ethically approved by the Animal Ethics Committee of Nanjing Medical University and adheres to ARRIVE guidelines. All methods were performed in accordance with the relevant guidelines and regulations. Mouse Interleukin 6 (IL-6), mouse Interleukin 17 (IL-17), and mouse Tumor Necrosis Factor α (TNF-α) ELISA KITs were bought from Wuhan CUSABIO Technology LLC.

### Synthesis of CS nanoparticles

In order to prepare the chitosan (CS) solution, 2.4 g of chitosan powder was gradually introduced into a solution of acetic acid (2%, 300 mL) at a temperature of 37 °C with stirring at a speed of 1080 rpm for a duration of 4.5 h. The resulting solution contained 0.8 wt% of chitosan and had a volume of 300 mL. A solution of TPP (0.1%, 200 mL) was then prepared by dissolving 0.2 g of TPP powder in 200 mL of ultrapure water. This TPP solution was added dropwise to the CS solution contained in a flask of 70 mL CS solution. The mixture was then stirred overnight at room temperature at a speed of 550 rpm. Following synthesis, the solution was centrifuged at a temperature of 4 °C, at a speed of 18,000 rpm for a period of 1 h. The pellet was subsequently washed with ultrapure water three times and finally resuspended in PBS for further analysis^56^.

### Synthesis of CSCE nanoparticles

As per the aforementioned steps, 2.4 g of chitosan powder was dissolved in a solution of acetic acid (2%, 300 mL) at a temperature of 37 °C through continuous stirring at a speed of 1080 rpm. Subsequently, 50 mg of ceftazidime powder was swiftly introduced into the 70 mL chitosan solution, and the mixture was stirred at a speed of 1000 rpm for a duration of 20 min at room temperature. This was followed by the gradual addition of 28 mL of TPP solution. The reaction was conducted at a stirring rate of 550 rpm, at room temperature. CSCE pellets were obtained via centrifugation of the reaction mixture at a temperature of 4 °C for a duration of one hour at a speed of 18,000 rpm. The supernatant was collected, and its absorbance value was measured. Meanwhile, the precipitate was resuspended in PBS for further analysis^57^.

### Ceftazidime loading and releasing assay

Following the synthesis of CSCE nanoparticles, the pellets were subject to centrifugation and washed with ultrapure water on multiple occasions. Subsequently, the supernatants were collected and their absorbance values were measured using a UV–Vis spectrometer at a wavelength of 257 nm to determine the concentration of ceftazidime (CE) in the CSCE nanocomposites. The CSCE nanocomposites were then separately resuspended in pH 7.4 and pH 6.4 PBS and subsequently incubated at a temperature of 37 °C for a duration of 24 h in a shaking incubator at a speed of 800 rpm. The supernatants were subsequently gathered, and their absorbance values were recorded at a wavelength of 257 nm. Utilizing Eqs. (1) and (2), respectively, the drug-loading capacity and -releasing percentage of the CSCE nanocomposites were calculated^58^.

1 $$ {\text{CE}}\,{\text{loading}}\,{\text{capacity}}\,\% { = }\frac{{{\text{total}}\,{\text{weight}}\,{\text{of}}\,{\text{CE }} - {\text{ CE}}\,{\text{in}}\,{\text{the}}\,{\text{supernatants}}}}{{{\text{weight}}\,{\text{of}}\,{\text{the}}\,{\text{CE}}}} \times 100. $$

2 $$ {\text{CE}}\,{\text{releasing}}\,{\text{percentage }}\,\% { = }\frac{{{\text{weight}}\,{\text{of}}\,{\text{CE}}\,{\text{in}}\,{\text{the}}\,{\text{supernatants}}}}{{{\text{weight}}\,{\text{of}}\,{\text{CE}}}}{ } \times 100, $$

### Minimal inhibitory concentration test

*Pseudomonas aeruginosa* PAO1 was propagated in LB medium at a temperature of 37 °C in a shaking incubator at 900 rpm overnight. Subsequently, 5 μL of the bacterial suspension was diluted in 1.2 mL of LB medium, after which 50 μL was added to the test wells of a 96-well plate. Different concentrations of CE (0.5, 0.25, 0.125, 0.0625, 0.0312, 0.0156, 0.0078, 0.0039, 0.0019 and 0.0009 mg/mL), CS (2.1, 1.68, 1.26, 0.84 and 0.42 mg/mL), and CSCE (1.0, 0.5, 0.2, 0.15 and 0.05 mg/mL) were then added individually into the wells. The mixture was subject to cultivation in a cell incubator at a temperature of 37 °C. Following 18 h of incubation, the wells not exhibiting bacteria under microscopy observation were considered as the minimal inhibitory concentration (MIC) of the samples^59^. All experiments were repeated three times in triplicate.

### Disk diffusion test (Kirby–Bauer test)

To assess the antibacterial properties of CS and CSCE nanoparticles, *P. aeruginosa* was cultured overnight in LB medium at 37 °C and 900 rpm in a shaking incubator. The bacterial solution was then diluted to 1 × 10^8^, 1 × 10^7^, and 1 × 10^6^ CFU/mL, and 100 μL was evenly spread on solid LB plates. To evaluate the antibacterial ability of the nanocomposites, circle filter papers with a 6 mm diameter were soaked individually in PBS (pH 7.4), 0.07 mg/mL of CE, 1.68 mg/mL of CS nanoparticles, and 1.75 mg/mL of CSCE nanoparticles for 30 min. The concentrations of the chitosan nanocomposites were determined by calculating the CE and CS concentration within the CSCE nanocomposites using their respective loading capacity (40 mg per gram) in the CS nanoparticles. The derived filter papers were placed on the solid LB plates and incubated at 37 °C for 20 h. The aseptic zones, which were observed as circular and clear zones on the bacterial lawn, were measured to determine the antibacterial abilities of the compounds contained in the filter papers^60^.

### Pyocyanin quantification assay

The suspension of *P. aeruginosa* PAO1, cultured overnight, was diluted to 0.4 A based on the OD_600_ value using LB medium. Subsequently, 1 mL of the bacterial suspension was mixed individually with different concentrations of CE (0.025, 0.125, and 0.0625 mg/mL), CS (0.56, 0.28, and 0.14 mg/mL), and CSCE (0.6, 0.3, and 0.15 mg/mL) and incubated at 37 °C and 900 rpm in a shaking incubator. These solutions were then centrifuged at 9000 rpm for 8 min, and 800 μL of the supernatants were subjected to pyocyanin extraction by mixing with 0.5 mL of chloroform. The derived chloroform was then incubated with 0.15 mL of 0.2 N HCL to transfer the colorless pyocyanin to a pink aqueous phase, and the absorbance values were measured at 520 nm using a microplate reader^36^ (Bio-Tek Instruments, Winooski, VT, USA).

### Hemolysis and degradation assay

To assess Hemolysis, 1 mL of whole blood from healthy individuals was added to 2 mL of PBS, and red blood cells (RBCs) were isolated via centrifugation at 400 rcf for 5 min. The RBCs were then washed three times with PBS and resuspended in 10 mL PBS. Subsequently, 0.2 mL of the RBC suspensions was incubated with CSCE in PBS at concentrations of 0.075, 0.15, 0.30, 0.60, and 1.19 mg/mL, while 0.8 mL of PBS and ultrapure water were used as negative and positive controls, respectively. The mixtures were gently stirred and incubated at 37 °C in a cell culture incubator for 2 h. Following centrifugation at 400 rcf for 5 min, absorbance values were recorded in a microplate reader at 490 nm to determine the amount of released Hemoglobin. The ratio of Hemoglobin release was calculated using Eq. (3). Moreover, 1.19 mg of CSCE nanoparticles was suspended in PBS (pH 7.4 and pH 6.4), and their morphology was observed via electron microscope after 72 h to ensure the degree of degradation. All experiments were repeated three times in triplicate^58,61^.

3 $$ {\text{Hemolysis}}\,{\text{releasing}}\,{\text{ratio }}\,\% { = }\frac{{{\text{experimental}}\,{\text{group}} - {\text{ negative}}\,{\text{control}}\,{\text{group}}}}{{{\text{positive}}\,{\text{control}}\,{\text{group}} - {\text{ negative}}\,{\text{control}}\,{\text{group}}}} \times 100. $$

### Biofilm inhibition and degradation assay

In our previous work, we addressed the biofilm inhibition and degradation assays. Briefly, the bacterial suspension was diluted to a specific ratio (0.2 OD_600_) using LB medium, and 100 μL of the resulting culture was mixed with 100 μL of PBS, CE (0.003125, 0.00625, 0.0125, 0.025, 0.05, 0.10 mg/mL), CS (0.07, 0.14, 0.28, 0.56, 1.12, 2.24 mg/mL), and CSCE (0.15, 0.30, 0.60, 1.20, 2.40, 4.80 mg/mL) in a 96-well plate. The concentrations of the nanoparticles were determined based on the ceftazidime-loading capacity of CSCE. After 48 h of incubation, the bacterial solutions were removed from the wells and washed thrice using PBS. The biofilms formed by *P. aeruginosa* were then fixed with 100% methanol for 15 min, washed three times with PBS, and stained with 0.5% crystal violet for 30 min at room temperature. After the removal of the dye solution, the wells were washed thrice with PBS and dried. Finally, anhydrous acetic acid was added to the wells, and the absorbance values were recorded at OD_570_ using a microplate reader^62^. In addition, to the degradation assay, *P. aeruginosa* PAO1 was cultured in LB medium in a 96-well plate for 48 h at 37 °C to form biofilms. Subsequently, 100 μL of PBS, ceftazidime, CS, and CSCE, used above, were added to the wells and incubated for 2 h at 37 °C in a pH 7.4 solution. The subsequent procedure was identical to the biofilm inhibition assay. Moreover, the *P. aeruginosa* PAO1 was incubated with PBS, CE (0.05 mg/mL), CS (1.14 mg/mL), and CSCE (1.19 mg/mL) at a specific concentration for 24 h to determine if the nanocomposites could attach to the bacterial membrane. The treated bacteria were then washed thrice with PBS and resuspended using 1 mL of 0.4% glutaraldehyde solution for overnight incubation at 4 °C. They were washed thrice with PBS and sequentially dehydrated with 20%, 50%, 80%, and 100% ethyl alcohol for 10 min each. Finally, 100% tertiary butanol was used to replace ethyl alcohol thrice, and the samples were aired for further observation under a scanning electron microscope^63^ (GeminiSEM 500 433, ZEISS, Germany).

### Cytotoxicity assay

The 293 T cells were cultured in DMEM medium and harvested at the exponential phase before being seeded in a 96-well plate with a density of 8000 cells per well. After incubation for 24 h, the cell culture medium was replaced with fresh medium containing either PBS or CSCE nanocomposites at concentrations of 0.075, 0.15, 0.3, 0.60, and 1.19 mg/mL. The cells were then cultured for an additional 48 h before adding 10 μL of CCK-8 solution to each well. The mixture was incubated in a cell incubator at 37 °C for 1–3 h. The cell viabilities in each well were calculated using Eq. (4) ^64^. All experiments were repeated three times in triplicate.

4 $$ {\text{Viability}}\,{\text{ratio}}\, \% { = }\frac{{{\text{experimental}}\,{\text{group}} - {\text{ blank}}\,{\text{group}}}}{{{\text{control}}\,{\text{group}} - {\text{ blank}}\,{\text{group}}}} \times 100. $$

### *P. aeruginosa* intraperitoneal infection model

To establish a *P. aeruginosa* i.p. infection model, we utilized 8-week-old ICR mice. Briefly, the PAO1 strain of *P. aeruginosa* was incubated in LB medium at 37 °C and 220 rpm for 18 h, followed by washing with sterile PBS solution. The bacterial pellets were resuspended in PBS and 1 × 10^7^ CFU/mL of *P. aeruginosa* PAO1 was injected into the lower left abdomen of each mouse using a 1 mL syringe after anaesthetizing them with 2% pentobarbital sodium. The mice were randomly divided into five groups with each group consisting of eight mice. At 6-, 12-, and 24-h post-infection, the mice were intraperitoneally injected with 200 μL of either PBS, ceftazidime, CS, or CSCE^65^. The dose of ceftazidime was 30 μg per gram of the mice while that of CS and CSCE was set at 19 μg and 23 μg per gram of the mice, respectively, according to the loading capacity of CSCE to ceftazidime. After five days, the mice were sacrificed by cervical dislocation and their bronchoalveolar lavage fluids were used to measure the concentrations of cytokines IL-6, IL-17, and TNF-α using Enzyme-linked Immunosorbent Assay (ELISA). The key organs such as the heart, liver, spleen, lung, and kidney were also removed and fixed in 4% paraformaldehyde overnight for H&E staining. A separate group of mice were used to evaluate the biocompatibility of CSCE nanoparticles. After an i.p. injection of either PBS, CE, CS or CSCE (n = 8), their organs were analyzed using H&E staining after being fixed with methanal for five days. To evaluate the biocompatibility of CSCE nanoparticles, we performed an in vivo assay using 8-week-old ICR mice that were unaffected. We injected either PBS, CE, CS or CSCE nanoparticles (n = 8) and after five days, their key organs were removed and fixed in methanal for H&E staining (n = 3). Before staining, these organs were dehydrated through alcohol reagents, embedded in paraffin wax, and then sectioned to 5 μm thickness. The samples were dewaxed in xylene, rehydrated through decreasing concentrations of ethanol, and washed in PBS. Next, each tissue was stained with hematoxylin and eosin followed by dehydration through increasing concentrations of ethanol and xylene. Finally, all sections were observed under an optical microscope to assess biocompatibility^66^.

### Statistical analysis

We conducted each experiment at least three times to ensure rigorous clinical analysis. We expressed the data as mean ± SD and used SPSS version 22.0 for statistical analysis. To test for differences between two groups, we performed two-tailed Student's t-tests with a *P*-value cut-off of 0.05 for statistical significance.

## Data Availability

The datasets used and/or analy**z**ed during the current study available from the corresponding author on reasonable request.
